# Choline PET/CT in Multiple Myeloma

**DOI:** 10.3390/cancers12061394

**Published:** 2020-05-28

**Authors:** Charles Mesguich, Cyrille Hulin, Axelle Lascaux, Laurence Bordenave, Gerald Marit, Elif Hindié

**Affiliations:** 1 CHU Bordeaux, Nuclear Medicine Department, F-33000 Bordeaux, France; laurence.bordenave@chu-bordeaux.fr (L.B.); elif.hindie@chu-bordeaux.fr (E.H.); 2 INSERM U1035, University of Bordeaux, F-33000 Bordeaux, France; gerald.marit@u-bordeaux.fr; 3 CHU Bordeaux, Haematology, F-33000 Bordeaux, France; cyrille.hulin@chu-bordeaux.fr (C.H.); axelle.lascaux@chu-bordeaux.fr (A.L.)

**Keywords:** multiple myeloma, PET/CT, ^18^FDG, ^18^F-Choline, ^11^C-Choline

## Abstract

The field of multiple myeloma (MM) imaging has evolved. The International Myeloma Working Group recently recommended performing ^18^F-fluorodeoxyglucose glucose (^18^FDG) positron emission tomography/computed tomography (PET/CT) with the aim of staging MM patients at baseline and evaluating response to therapy. Novel oncological radiotracers such as ^11^C-Choline and ^18^F-Fluorocholine, have been studied in comparison with ^18^FDG, mostly in MM patients presenting with refractory disease or suspected relapse. Choline-based tracers may overcome some limitations of ^18^FDG, which include a lack of sensitivity in depicting skull lesions and the fact that 10% of MM patients are FDG-negative. The majority of MM lesions display a higher uptake of Choline than FDG. Also, in many situations, Choline may offer better lesion visualization, with a higher tumor to background ratio; however, various patterns of Choline and FDG uptake have been observed in MM and some limitations, notably as regards liver lesions, should be recognized. Overall, Choline may provide additional detection of up to 75% more lesions. This article aims to provide a comprehensive review of the potential role of Choline in multiple myeloma, as compared to FDG, encompassing Choline physiopathology as well as data from clinical studies.

## 1. Introduction

Multiple myeloma (MM) imaging has rapidly evolved during the past few years. Functional imaging such as magnetic resonance imaging (MRI) and ^18^F-fluorodeoxyglucose glucose (^18^FDG) positron emission tomography/computed tomography (PET/CT) are now recommended for baseline staging by the International Myeloma Working Group (IMWG) [1]. Additionally, the IMWG recently recognized the prognostic value of ^18^FDG PET/CT in the evaluation of treatment response [2,3,4].

^18^F-Fluorocholine (FCH) and ^11^C-Choline are PET/CT radiopharmaceuticals that were initially developed for prostate cancer imaging [5]. Indeed, Choline PET/CT can help to locate anatomical sites of disease recurrence when there is biochemical suspicion of relapse. Choline PET/CT is also recommended for the staging of well-differentiated hepatocellular carcinoma [6]. Choline-based tracers are emerging radiopharmaceuticals in the field of MM and could offer several advantages over ^18^FDG. This article aims to provide a comprehensive review of the potential role of Choline in MM, as compared to FDG, encompassing Choline physiopathology as well as data from clinical studies.

## 2. ^18^F-Choline and ^11^C-Choline Physiopathology

FCH and ^11^C-Choline have similar biodistributions [5]. Choline is a precursor to the synthesis of phospholipids [7]. It is internalized by the cell through a choline transporter and is then transformed into phosphocholine by a Choline Kinase. It is then coupled to diacylglycerol to form phosphatidylcholine, a major component of cell membranes; therefore, Choline is thought to reflect the intensity of cell membrane synthesis. 

Choline can be labeled either with ^11^C or ^18^F. Even though ^11^C has the advantage of yielding a natural compound, the use of ^11^C-Choline is limited due to ^11^C short half-life of 20 minutes, which necessitates the presence of an on-site cyclotron. On the other hand, the longer half-life of ^18^F (110 min) allows FCH to be distributed to PET centers that are distant from the production site. 

After injection, ^11^C-Choline is rapidly cleared from the blood and optimal tumor-to-background contrast is reached within 5–7 min [8,9]. Choline is mainly metabolized by the liver, the main organ for lipid metabolism. Intense uptake in the liver (mean SUVmax of 11.4) and pancreas (mean SUVmax of 7.8) is observed [10]. Moderate uptake is seen in the spleen, salivary glands, and lachrymal glands. Mild uptake can be observed in the small and large intestines, breasts, and testicles as well. Bone marrow uptake is usually low (mean SUVmax of 1.7) [10], similar to ^18^FDG (SUVmean of 1.7 at the lumbar spine [11]). There is no relevant uptake in the adult brain, except in the choroid plexus and the pineal gland. ^11^C-methyl-choline can be oxidized to betaine by choline oxidase, with detectable metabolites soon after radiotracer injection [12]. Due to Choline urinary excretion, there is also an intense activity in the kidneys and the bladder. FCH has the advantage of having less urinary excretion than ^11^C-Choline.

Malignant tumors can exhibit a high choline uptake due to their high rate of replication, implying a high turnover of the cell membrane. Besides cell replication, other metabolic pathways may be involved in choline uptake, as it seems not always related to the levels of Ki67 expression [13]. For example, Choline can be involved in the synthesis of acetylcholine and can also be metabolized to betaine [14]. Pathological Choline uptake is also not specific to cancer. Indeed, Choline uptake can be observed in cases of inflammatory conditions, as the monocyte-to-macrophage differentiation implies a high rate of cell membrane synthesis and lipogenesis. Hence, reactive lymph nodes including granulomatous disease can be a major cause of interpretation pitfalls [10].

The uptake of Choline by MM cells might be related to different mechanisms. First, choline uptake can be related to the rate of replication of clonal plasma cells. Second, choline uptake may be linked to lipogenesis, as fatty acids and lipids play an important role in the pathogenesis of MM cells. Lysophospholipid levels (lysophosphatidylcholine and lysophosphatidic acid) are indeed increased in MM patients compared to healthy subjects [15]. Fatty acid synthetase expression is also upregulated in myeloma cells, contributing to proliferation and survival [16]. In relapsed and in high-risk MM, phosphatidylcholine is downregulated and is thought to be hydrolyzed to form lipid messengers, responsible for tumor dissemination [17,18]. Hence, MM cells may exhibit different patterns of uptake depending on the patient’s previous exposure to MM drugs.

## 3. Choline PET/CT vs. ^18^FDG PET/CT in Multiple Myeloma

^18^FDG is a widely used PET radiotracer offering diagnostic and prognostic information in many hematological and solid cancers. It reflects the level of glycolytic activity of cancer cells, which is increased in about 90% of newly diagnosed MM [19]. ^18^FDG PET/CT offers a thorough evaluation of MM tumor burden at diagnosis and carries a powerful prognostic value at baseline and during therapy response assessment [20,21,22,23,24].

Bellow, we discuss the findings relating to the detection of focal bone lesions and extra-medullary disease by ^18^FDG and Choline PET/CT.

### 3.1. Focal Bone Lesion Detection

The comparison of Choline PET/CT and ^18^FDG PET/CT lesion detectability in MM has been performed in the past few years (Table 1). In a study conducted by Nanni and colleagues [25], 10 patients underwent both ^11^C-Choline and ^18^FDG PET/CT scans within one week. The settings were: evaluation after completion of initial therapy (*n* = 4), disease relapse (*n* = 4) and follow-up (*n* = 2). Six patients were positive on both examinations, with a total of 37 bone lesions detected on ^11^C-Choline PET/CT vs. 22 lesions on ^18^FDG PET/CT. Four patients had concordant negative findings. The last patient had only one lesion in the pelvis, which was ^18^FDG-positive but ^11^C-Choline negative. None of the patients had a positive ^11^C-Choline PET combined with a negative ^18^FDG PET. Choline intensity of uptake was globally superior to that of FDG, with a mean SUVmax of 5.0 vs. 3.8 respectively (*p* = 0.042); however, it is interesting to note that some lesions had a high ^18^FDG uptake but a low Choline uptake. Moreover, a mix of uptake patterns of MM lesions could be observed within the same patient. 

In a second study by Cassou-Mounat et al. [26], 21 MM patients underwent FCH-PET/CT and FDG PET/CT within a median time of 7 days, for progression under treatment (*n* = 6) or suspected relapse (*n* = 15). Nineteen were biochemically confirmed. One patient was detected as positive for bone involvement by FCH-PET/CT only, while no patient was positive by FDG PET/CT only. After masked reading, the mean number of foci per patient was 4.6 for FDG vs. 8.1 for FCH (*p* < 0.001) with a total number of 69 lesions for FDG and 121 for FCH. Among the 69 FDG-positive lesions, 65 were also FCH-positive. The 56 foci that were FCH+/FDG- were predominantly located in the skull and the torso. The median SUVmax values for the FCH-detected lesions (3.8 and 5.7) were higher than for the FDG-detected lesions (3.0 and 4.5). Additionally, the tumor/background ratio of Choline was superior to that of FDG. 

**Figure 1:**
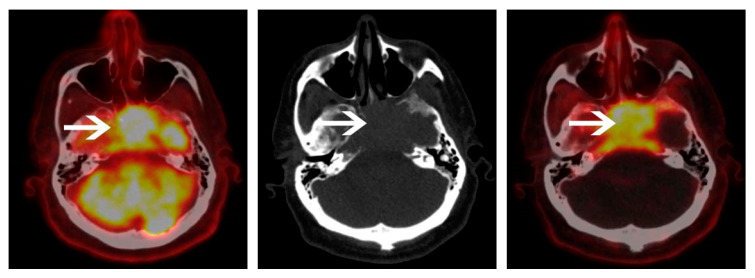
A 65-year-old male with a light chain multiple myeloma (MM). ^18^F-fluorodeoxyglucose glucose (^18^FDG) positron emission tomography/computed tomography (PET/CT) (left panel) and ^18^F-Choline PET/CT (right panel) were performed with a 4-day interval. ^18^F-Choline PET/CT axial image of the skull shows an intense uptake of a skull base lesion. Because of the intense surrounding cerebral uptake on ^18^FDG PET/CT, the lesion is more difficult to individualize. Clinical examination at baseline identified the presence of diplopia and left ptosis, which disappeared a few days after induction chemotherapy was started.

**Figure 2:**
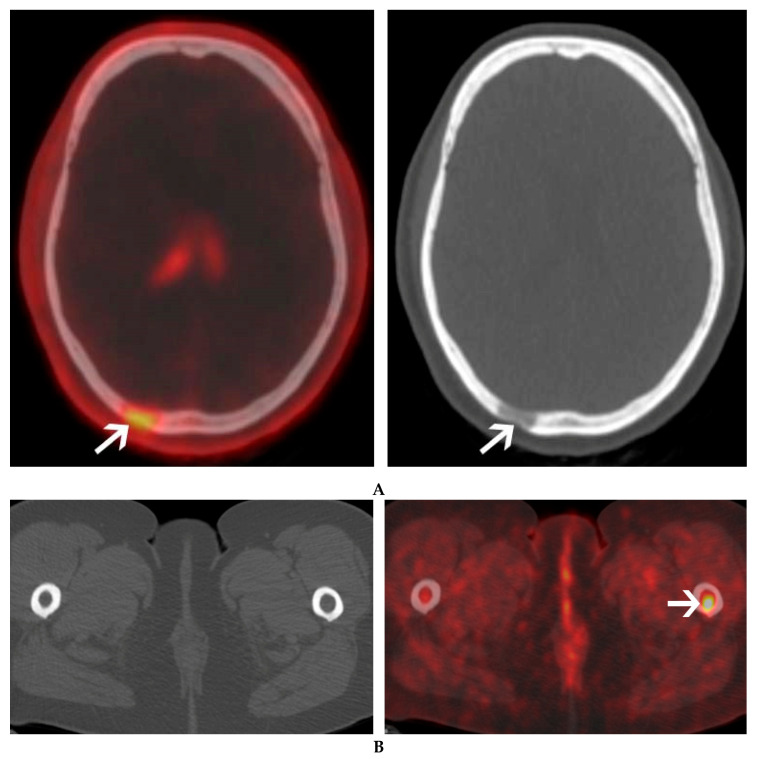
A 60-year-old female with IgG lambda smoldering multiple myeloma. An osteolytic lesion of the skull was found on a follow-up CT. ^18^F-Choline PET/CT was ordered to further characterize this lesion and search for additional bone lesions. ^18^F-Choline PET/CT axial image of the skull shows a moderate uptake of an occipital osteolytic lesion (**A**). Additional focal uptake of ^18^F-Choline was seen in the left femur, corresponding to a bone marrow lesion with no bone structural changes on CT (**B**).

Overall, in relapsed/refractory MM, Choline PET/CT detects up to 75% more bone lesions than ^18^FDG PET/CT [25,26]. Choline also offers better visualization of MM lesions than FDG, which can contribute to a higher reading comfort but also higher inter-observer agreement. The low sensitivity of FDG for skull lesions is explained by the high glucose uptake in adjacent brain tissues (Figure 1). This limitation is overcome by Choline tracers (Figure 2). However, the high liver uptake of Choline may limit its sensitivity to depict lesions of surrounding bone areas such as the right costal grid.

### 3.2. Uptake Patterns of 18FDG and Choline-Based Tracers

**Figure 3:**
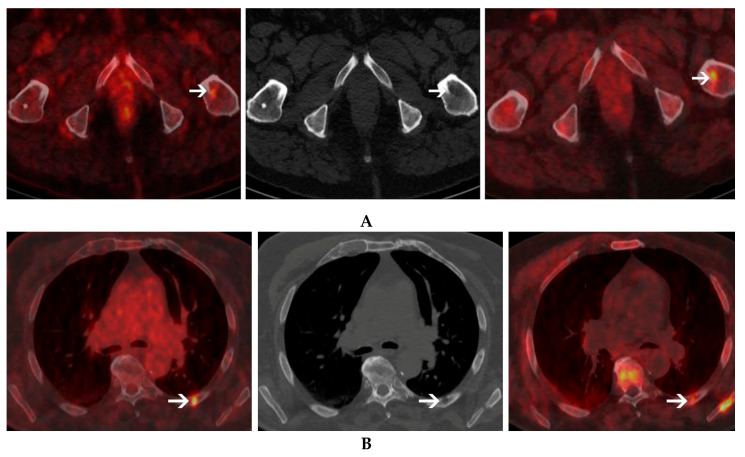
A 72-year-old male with light chain multiple myeloma. ^18^FDG PET/CT (left panel) and ^18^F-Choline PET/CT (right panel) were performed with a 5-day-interval. Different uptake patterns are seen in this patient. Lesion exhibiting high choline uptake but low FDG uptake can be seen in the left femur (**A**). In contrast, however, a lesion with high FDG uptake but low Choline uptake can be seen in the posterior arch of the left 6th rib (**B**) corresponding to an osteolytic lesion on CT (central panel).

**Figure 4:**
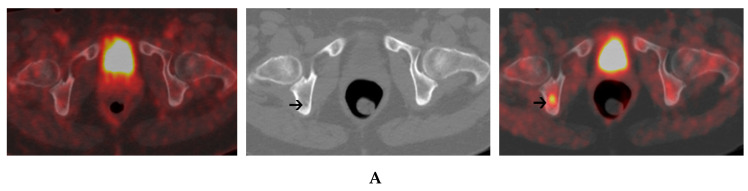

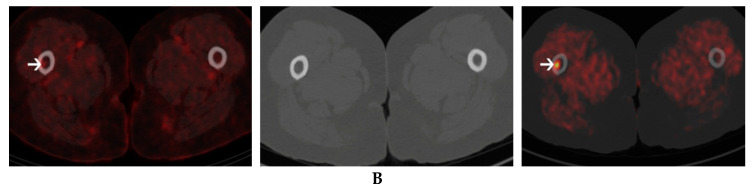
A 56-year-old female with IgG kappa MM. ^18^FDG PET/CT (left panel) and ^18^F-Choline PET/CT (right panel) were performed with a 3-day-interval. An axial PET/CT image of the pelvis shows a focal uptake of ^18^F-Choline within the right ischium without a corresponding uptake on ^18^FDG PET/CT. The corresponding axial CT image (central panel) shows no clear-cut structural changes (**A**). A focal lesion of the right femur exhibits a high uptake of ^18^F-Choline. This lesion exhibits only a faint uptake on ^18^FDG PET/CT (left panel) and is not visible on CT (central panel) (**B**).

A difference in uptake pattern between ^18^FDG and Choline can be observed among patients but also within the same patient (Figure 3) [25,26]. FDG_high_/Choline_low_ uptake of MM lesions are relatively rare. One needs first to exclude false-positive findings of FDG, with for example recent fractures that can bring substantial inflammation leading to increased FDG uptake [26]. MM patients are prone to bone fractures, especially of the ribs and spine, as these patients are at higher risk of having osteoporosis [28]. FDG_high_/Choline_low_ uptake can be seen in aggressive or “dedifferentiated” lesions [26]. This uptake pattern has already been documented in hepatocellular carcinoma and is a signature of aggressive disease [29]. The more frequently observed pattern of Choline_high_/FDG_low_ uptake of MM lesions may have different causes. It may correspond to early lesions, as only a minority of these foci were accompanied by osteolytic changes on the corresponding CT images (35%) [26] (Figure 4). It may also reflect a more indolent form of the disease [26]. It was recently pointed out that about 10% of newly diagnosed MM patients, with bone lesions on MRI, are FDG-negative, which is linked to a lower expression of hexokinase-2 [19]. Another study showed that newly diagnosed MM patients with false-negative findings on FDG PET/CT had longer PFS than FDG-positive patients [30]; however, other mechanisms also appear to be involved in the uptake as FDG-negative patients with relapsed/refractory disease were found to have normal expression of Hexokinase-2 [31]. Finally, the use of dexamethasone as a treatment option can decrease FDG-uptake by promoting MM cell apoptosis and by lowering the inflammatory environment of MM cells. Overall, these results corroborate the recent findings of multiple, spatially separated clones that coexist in the same patient, carrying different genotypic mutations [32]. These clones can be individualized even in patients with negative minimal residual disease. This highlights the complementary role of whole-body imaging, and the possible advantage of using more than one tracer in some situations that remain to be defined. Whether these different patterns are linked to patient prognosis should also be investigated in prospective studies.

Although a good correlation can be observed between Choline uptake and plasma cell infiltration on BMB (R^2^ = 0.52) [27], the performance of Choline for the diagnosis of diffuse infiltration pattern has not yet been addressed. Studies prospectively comparing MRI and Choline PET/CT in that regard are warranted.

### 3.3. MYELOCHOL: A Prospective Pilot Study

The MYELOCHOL study is a prospective monocentric study that is aimed at comparing FCH and ^18^FDG PET/CT performance in newly diagnosed MM. Both of the previous studies performed have a common limitation [25,26]. Lesions were assessed without a pre-determined reference standard able to differentiate between true-positive and false-positive findings. MRI is the gold standard for bone marrow imaging [33], which makes it a good candidate for Choline PET/CT performance evaluation, as obtaining biopsy results for each lesion would not be feasible. Additionally, the performance Choline PET/CT is yet to be evaluated in a patient population that has not been exposed to MM drugs. The treatment of MM may indeed select clones that could behave differently from baseline [32].

The MYELOCHOL study plans to include 30 newly diagnosed MM patients (Haut-Lévêque Hospital, University of Bordeaux, Bordeaux, France). All patients will undergo Whole-Body MRI, FCH, and 18FDG PET/CT with a maximal interval of 7 days between each imaging modality. Whole-Body MRI will be the standard of reference. Each imaging study will be read blindly by two nuclear medicine physicians with expertise in MM. A second step will include a comparative reading of each PET lesion with MRI findings, to determine if the lesion is a true positive or a false positive. In case of a Choline-positive lesion not found on MRI, a multidisciplinary team, composed of nuclear medicine, radiology, and hematology physicians, will come to a consensus as to whether the lesion can be related to MM. Study enrollment started in September 2019 and is currently ongoing.

### 3.4. Extra-Medullary Disease

Multiple myeloma patients with extra-medullary disease (EMD) have a worse prognosis [34]. Extra-medullary lesions develop in any soft tissue and should be differentiated from soft tissue lesions expanding from adjacent bones (“breakout lesion”) [35]. In line with what was found for bone lesions, Choline uptake within EMD can either be higher or lower than FDG-uptake [25,26]. The detectability of liver EMD lesions may be hampered due to the high physiological choline uptake by the liver. Moreover, benign hepatic lesions such as nodular hyperplasia may mimic MM EMD [6]. Generally, caution must be taken when diagnosing EMD with Choline or FDG, as inflammatory or neoplastic processes are known confounders for both tracers [10,36].

### 3.5. Therapy Response Assessment

The value of Choline PET/CT for response assessment in MM has not yet been explored. The IMPETUS criteria have been defined for harmonizing ^18^FDG PET/CT readings with the use of the Deauville five-point scale [37]. This scale implies the use of liver uptake as a reference above which a lesion could be considered positive after treatment; however, Choline-specific criteria will have to be elaborated for response evaluation, mainly because of the markedly intense liver uptake of this tracer. 

The main features of Choline PET/CT and FDG PET/CT are summarized in Table 2

## 4. ^11.^ C-Choline PET/CT vs. ^11^C-Methionine PET/CT and Other PET Tracers

Lapa and colleagues investigated the detectability rate of ^11^C-Choline compared to ^11^C-Methionine [27]. Nineteen pre-treated patients received both PET/CT scans within a median time of 10 days. No reference standard was used. Fifteen patients had positive findings, with only one patient with a methionine positive PET/CT but negative choline PET/CT. Choline and Methionine detected an equal number of lesions in 11/19 patients while Methionine detected more lesions in 8/19 patients. Seven patients had disseminated disease (>50 FL) that was identified by both tracers in four patients. In the remaining three patients with disseminated disease on Methionine PET/CT, Choline PET/CT only identified a total number of four lesions. The uptake of Methionine was superior to that of Choline with a median SUVmax of 9.3 (3.5–39) vs. 5.7 (3.5–14.6), respectively. A better tumor/background ratio was also observed with ^11^C-Methionine (18.7 vs. 8.8, *p* = 0.0001). Although ^11^C-Methionine appears to yield better results than ^11^C-Choline, its availability is currently limited, considering the difficulties related to the short half-life of carbon-11.

Besides FDG, Choline, or Methionine, other radiotracers are of potential interest, with especially radiopharmaceuticals that can be used for disease imaging and treatment as well, constituting important theranostic applications [38], such as radiolabeled agents targeting the prostate-specific membrane antigen (PSMA), the chemokine receptor CXCR4 [39] or radiolabeled CD38-targeting antibodies [40,41]. A cluster of differentiation CD38 is highly expressed on myeloma cells. Daratumumab is the first fully human monoclonal antibody targeting CD38 for the treatment of MM. It improves the depth and duration of response in combination with other antimyeloma agents [42,43]. Daratumumab has been successfully labeled with ^89^Zr and ^64^Cu with promising immuno-PET imaging results in pre-clinical studies but also recently in small in-human studies [40,41]. CD38-targeted radioimmunotherapy has also been investigated preclinically [44,45]. The rate of patients with high CD38 expression will determine how many of them could beneficiate from anti-CD38 immuno-PET imaging and possibly CD38-targeted-immunoradiotherapy. 

## 5. Conclusions

In multiple myeloma, PET/CT performed with Choline-based tracers may offer several advantages over ^18^FDG PET/CT, showing better detectability of focal bone lesions; however, the diagnostic performance of Choline PET/CT should be further explored in larger prospective studies, which should be designed with a reference standard. These data are required before clinical routine implementation can take place. Studies focusing on the prognostic value of Choline PET/CT and its value as regards treatment response evaluation are also warranted.

## Figures and Tables

**Table 1:** Characteristics and main results of studies exploring the use of ^11^C-Choline or ^18^F-Choline in multiple myeloma.

References	Number of Patients	Study Design	Time of Imaging	Index Test	Choline Administered Activity	Choline PET Scanning Time Post-Injection	Reference Test	Number of Bone FL on Choline PET/CT (Matched/ Unmatched)	Number of Bone FL on FDG- or MTH PET/CT (Matched/ Unmatched)	Number of EMD foci on Choline PET/CT (Matched/ Unmatched)	Number of EMD foci on FDG- or MTH PET/CT (Matched/ Unmatched)	Mean or Median (range) SUVmax of Choline PET	Mean or Median (range) SUVmax of FDG- or MTH PET
**Nanni et al. (2007)** [25]	10	Retrospective	Relapse or f/up post treatment	^11^C-Choline ^18^F-FDG	5.3 MBq/kg	5 min.	None	37 (21/16)	22 (21/1)	3 (2/1)	1 (1/0)	5.0 (NS)	3.8 (NS)
**Cassou-Mounat et al. (2016)** [26]	21	Retrospective	Relapse or Progression	^18^F-Choline ^18^F-FDG	3 MBq/kg	10–20 min.	None	121 (65/56)	69 (65/4)	3 (3/3)	3 (3/3)	3.8-5.7 (1.6–16)	3-4.5 (1.6–20.6)
**Lapa et al. (2019)** [27]	19	Retrospective	Progression or f/up post-treatment	^11^C-Choline ^11^C-MTH	719 ± 86 MBq	5 min.	None	91* (91/0) >50: 4 patients	112 ** (87/25) >50: 7 patients	0	0	5.7 (3.5–14.6)	9.3 (3.5–30.9)

MTH: Methionine; FL: Focal bone Lesion; EMD: Extra-Medullary-Disease; NS: Not Specified *91 FL were found in 15 patients. The remaining four patients had innumerable FL (>50) which were all matched by MTH PET/CT. ** 112 FL were found in 12 patients. The remaining 7 patients had innumerable FL (>50) with 3 of them having less than 3 lesions on Choline PET/CT.

**Table 2:** Comparative table of the main characteristics of ^18^F-Choline or ^11^C-Choline PET/CT vs. ^18^FDG PET/CT in multiple myeloma.

Characteristics	^18^F or ^11^C Choline PET/CT	^18^FDG PET/CT
Cost	>^18^FDG	<^18^F or ^11^C Choline
Availability	<^18^FDG	Widely available
Delay between injection and acquisition	5–20 min (shorter than with ^18^FDG)	60 min.
Tumor/Background ratio	>^18^FDG	<^18^F or ^11^C Choline
Detectability of MM lesions	Up to 70% more lesions than ^18^FDG	10% of MM are ^18^FDG-negative
Areas of impaired detection	-FL of right lower ribs: “liver shadowing” -Liver EMD	Skull FL: “brain shadowing”
Effect of Dexamethasone on uptake	Unknown	Impaired uptake
Correlation with bone marrow infiltration	Good (R^2^ = 0.52)	Moderate (R^2^ = 0.36)
False-positive findings	Granulomatosis, neoplasia, inflammatory processes	Neoplasia, inflammatory processes, granulomatosis
Prognostic value	Unknown	Proved in multiple studies at baseline and during therapy response assessment
Interpretation criteria	Undefined	IMPETUS criteria

MM: Multiple Myeloma; FL: Focal Lesion; EMD: Extra-Medullary Disease; IMPETUS: Italian Myeloma Criteria for PET Use.
